# Systemic inflammatory response syndrome in patients with acute obstructive upper tract urinary stone: a risk factor for urgent renal drainage and revisit to the emergency department

**DOI:** 10.1186/s12894-020-00644-z

**Published:** 2020-06-29

**Authors:** Spencer Larkin, Jeremy Johnson, Trisha Venkatesh, Joel Vetter, Ramakrishna Venkatesh

**Affiliations:** 1 Department of Urology, University of Kentucky, Lexington, KY USA; 2 Washington University in St. Louis, St. Louis, MO USA; 3 Division of Urology Surgery, Washington University School of Medicine, 4960, Children’s place, Campus Box 8242, St. Louis, MO 63110 USA

**Keywords:** SIRS, Urolithiasis, Renal obstruction, Emergency department, Ureteral colic

## Abstract

**Background:**

In patients seen in the emergency department (ED) with acute stone obstruction many risk factors that indicate need for urgent renal drainage are known. However, in patients discharged from ED without renal drainage factors that can minimize revisit to the emergency department are not fully identified. We evaluated SIRS (systemic inflammatory response syndrome) as a risk factor for urgent renal drainage and revisit to the ED in patients with acute stone colic during their ED visit.

**Methods:**

Retrospective review was performed of patients presenting to a tertiary academic emergency department (ED) from an obstructing ureteral or UPJ stone with hydronephrosis confirmed on an abdominal and pelvic CT scan. Data evaluated over a 3-year period included stone size, presence of UTI, presence or absence of SIRS and other clinical variables as risk factors for urgent renal drainage and ED revisits.

**Results:**

1983 patients with urolithiasis were seen at the ED and 649 patients had obstructive urolithiasis on CT scan. SIRS was diagnosed in 15% (99/649) patients. 54/99 (55%) patients with SIRS underwent urgent renal drainage compared to 99/550 (17%) in non-SIRS patients. In a multivariate analysis SIRS was a predictor of urgent intervention compared to non-SIRS patients (odds ratio 4.6, *p* < 0.05). SIRS was also associated with increased risk for revisits to the ED (6.9% with SIRS vs. 2.4% with no SIRS, odds ratio 2.9, *p* = 0.05).

**Conclusions:**

Presence of SIRS in obstructive urolithiasis patients was an independent risk factor of acute urologic intervention and revisits to the ED. A timely consultation with a urologist following discharge from ED for obstructive stone patients with SIRS who had no acute renal drainage may prevent revisit to the ED. Evaluation for SIRS in addition to other clinical risk factors should be considered while making management decision in patients with acute stone obstruction.

## Background

Obstructive urolithiasis is a common problem resulting in nearly 1.2 million ED visits per year in the United States [1]. A study using a nationwide emergency department sample, the largest all-payer ED database in the United States, reported an average of 19% hospital admission rate for patients presenting with ureteral colic [1]. With high prevalence rates, hospital admissions and enormous inpatient costs, identifying risk factors that affect patient revisit to the ED can help making improvements towards an efficient and safe management of ureteral stone colic patients presenting to the emergency department.

A previous study identified some of the risk factors associated with the need for acute urological intervention in patients presenting with ureteral colic [2]. These included stone size of ≥5 mm, proximal ureteral stone, presence of nitrites or leucocyte esterase on chem-strip urinalysis, age over 50 years, tachycardia at triage and abnormal serum WBC count. The above factors were independently associated with urologic intervention within 90 days in renal colic patients presenting to the ED. Another study reported on the association of pregnancy, diabetes and presence of urinary infection with higher hospital admission rates from the ED and greater cost [1]. In our cohort we studied risk factors at the initial evaluation in the emergency room including SIRS for an urgent need for renal drainage and patient disposition in patients presenting with an obstructive ureteral or UPJ stone. For patients who revisit the ED, pain is the most common variable in addition to symptoms of uti.

Systemic inflammatory response syndrome (SIRS) has been used by many clinicians as a predictor of early sign of severe sepsis in the ICU and the ED settings [3, 4]. The newer sepsis prediction assessment qSOFA (quick Sequential Organ Failure Assessment) has been well studied in an ICU setting but not so much in an ED setting. In a comparative study on predicting mortality from sepsis in an ED setting, qSOFA has shown to be less sensitive compared to SIRS, although it has better specificity [5]. The significance of the presence of SIRS in patients presenting to the ED with obstructing stone has not be previously reported. This includes questions such as ‘in the presence or absence of SIRS how safe is it to discharge patients from ED with obstructing stones’. Studying SIRS during the early evaluation process in the ED may help with better management of obstructive stone patients including predicting the need for urgent urologic intervention, minimizing ED revisits and plan proper patient disposition. In the above cohort we hypothesized that presence of SIRS impacts the need for urgent renal drainage and revisit to the ED in patients presenting with an obstructing urinary stone.

## Methods

After obtaining the University of Kentucky IRB approval, we performed a retrospective chart review of patients at our tertiary care academic emergency department. We identified patients using ICD-9 codes 592.0 (calculus of kidney), 592.1 (calculus of ureter) and 592.9 (urinary calculus, unspecified) and a database query was performed for 3 years (2013–2016) of adult patients presenting to the ED and included all patients that had ureteral stone or UPJ stone and hydronephrosis on CT scan. We excluded all patients who did not have a CT scan available, patients who did not have all the SIRS criteria recorded and all pediatric patients (< 18 years old). Data collected included age, gender, vital signs, WBC count, stone size, presence of urinary infection, disposition (home, inpatient admission, operating room visit for ureteral stenting), readmission to the ED and subsequent surgical intervention. The concept of SIRS was developed in 1992 and the goal was to identify risk factors for the development of severe sepsis by using common, easy-to-use clinical parameters [1]. There are four components of SIRS (temperature < 36°or > 38 °C, heart rate > 90 bpm, respiratory rate > 20 per minute, WBC# < 4000 or > 12,000 cells/mm^3^) and patients with SIRS meet at least two of these criteria. SIRS in the presence of a source of infection is sepsis. Severe sepsis is sepsis with one or more organ dysfunction. Septic shock is hypotension despite adequate fluid resuscitation along with perfusion abnormalities. We evaluated patients for the presence of UTI on urine dipstix test and urine culture, patients with sepsis (SIRS with infection), and septic shock. We also compared the outcomes in patients with and without SIRS, including positive and negative for urinary infection on dipstix test and culture. Of note, we followed as best we could, the checklist for best practice of reporting observational studies as per STROBE (The Strengthening the Reporting of Observational Studies in Epidemiology) statement [6].

Univariate and multivariate analyses was performed. A *p*-value of < 0.05 was considered statistically significant. For univariate analysis, Fisher’s exact test was used for qualitative data and Wilcoxon sum-rank test for quantitative data. Multivariate logistic regression models were used to calculate the odds of patients needing an operative procedure and ED revisits with case wise deletion used for missing data. All analysis was performed using R version 3.2.2.

## Results

A total of 5002 patients with urolithiasis were identified who visited ED that had urolithiasis among other diagnosis codes. Of these, 1983 patients were included because of their primary diagnosis was either ICD-9 code 592.0, 592.1 or 592.9. Chart reviews were performed on these 1983 patients and 649 patients were identified as having a ureteral stone or UPJ stone with hydronephrosis on CT scan and the required SIRS criteria data available. Majority of patients (77%) from ED were discharged and 23% underwent urgent renal drainage.

SIRS was present in 15% (99/649) of patients seen at the ED with obstructive urolithiasis. Only 45% of patients with SIRS were discharged from ED compared to 83% of non-SIRS patients. Over half of SIRS patients (54/99, 55%) underwent emergent surgical renal drainage compared to 17% (94/550) of non-SIRS patients. Of patients discharged home, 13% of SIRS patients and 8% of non-SIRS patients returned to ED. Clinically significant factors that predicted patients going to OR for ureteral stenting were larger stone size (7 mm vs. 4 mm, *p* < 0.001), presence of SIRS (36% vs. 9%, p < 0.001), presence of UTI (14% vs 0.3%, p < 0.001). Table 1 represents on uni and multivariate analysis the odds ratio of independent predictors requiring urgent renal drainage. Not surprisingly, sepsis had the highest odds ratio of 28, followed by urine dipstick positive for infection (odds ration of 5.5), presence of SIRS (odds ratio 4.6), and stone size (odd ratio 1.4). Among the SIRS criteria, WBC count, elevated temperature and HR had increased odds ratio of going to the operating room. Of note, all our patients in the above cohort underwent ureteral stenting for stone obstruction due to local hospital practice pattern and none had percutaneous nephrostomy. Patients with SIRS compared to non-SIRS patients are more likely to be admitted to hospital, undergo surgical intervention, had UTI (odds ratio 6.4). In our cohort presence of SIRS was indicative of UTI compared to those without SIRS. SIRS patients were also more likely to go into severe sepsis or septic shock, but the number of patients was small to make valid conclusions.

**Table 1:** Univariate and multivariate analysis of factors for urgent renal drainage

	Univariate Table			Multivariable Model			
Variable	Urgent Drainage (No)	Urgent Drainage (Yes)					
	Mean (SD) or N (%)	Mean (SD) or N (%)	***p***-value	Odd’s Ratio	95 Lower	95 Higher	***p***-value
n	501	148					
Age	41 (15)	46 (16)	0.003	1.02	1.00	1.03	0.026
Male	322 (64)	60 (40)	< 0.001	0.40	0.26	0.63	< 0.001
SIRS	45 (9)	54 (36)	< 0.001	4.63	2.65	8.09	< 0.001
Sepsis	1 (0.2)	21 (14)	< 0.001	28.47	3.57	226.99	0.002
Stone Size	4 (2.0)	7.3 (6)	< 0.001	1.40	1.28	1.53	< 0.001
Temperature (Celsius)	37 (0.3)	37 (0.6)	< 0.001	2.32	1.31	4.11	0.004
HR	79 (15)	92 (21)	< 0.001	1.03	1.01	1.04	< 0.001
RR	19 (3)	19 (3)	0.074	1.07	0.98	1.16	0.122
WBC#	10 (3)	13 (13)	< 0.001	1.16	1.09	1.23	< 0.001

Table 2 represents patients with and without SIRS with odds ratios of different variables. Patients with SIRS had higher odds of hospital admission, surgical intervention, presence of UTI and revisit to the ED. Of note, patients with SIRS with negative urine dipstix for infection had lesser incidence of surgical intervention (23%) compared to those who had SIRS with positive dipstix (73%). Of note, 7% of patients with SIRS who were discharged from the ED had revisits to the ED compared to 2% of patients with no SIRS that were discharged from the ED (*p* = 0.05).

**Table 2:** Individual Cohorts and Characteristics of SIRS and Non-SIRS patients where urine dipstix data was available (483 patients)

	Non- SIRS	SIRS	Overall	Odds Ratio for SIRS vs. Non-SIRS	***P***-value
**No. patients**	411	72	483		
**Age (years)**	41 (16–91)	44 (22–94)	42 (16–94)	1.01 (0.99–1.03)	0.309
**Male (%)**	247 (60)	37 (51)	284 (59)	0.70 (0.42–1.16)	0.194
**Temp. (Celsius)**	37 (36–38)	37 (36–39)	37 (36–39)	2.30 (1.65–3.21)	0.002
**Heart Rate (beats/min)**	78 (23–142)	104 (59–153)	82 (23–153)	1.09 (1.07–1.12)	< 0.001
**Resp. Rate (breaths/min)**	18 (10–28)	21 (15–36)	19 (10–36)	1.35 (1.23–1.49)	< 0.001
**WBC # (cells/mm**^**3**^**)**	10 (2–159)	14 (6–41)	11 (2–159	1.10 (1.04–1.16)	< 0.001
**Stone Size (mm)**	5 (1–26)	5 (2–30)	5 (1–30)	1.05 (0.98–1.13)	0.521
**D/C’d Home**	341 (83)	35 (49)	376 (78)	0.19 (0.11–0.33)	< 0.001
**To OR on 1st ED visit**	64 (16)	33 (46)	97 (20)	4.59 (2.69–7.83)	< 0.001
**Readmitted**	10 (2)	5 (7)	15 (3)	2.99 (0.99–9.03)	0.057
**Infection source identified**	96 (23)	47 (65)	143 (30)	6.17 (3.61–10.55)	< 0.001
**Proven infection present**	60 (15)	35 (49)	95 (20)	5.53 (3.23–9.47)	< 0.001
**UTI present**	53 (13)	35 (49)	88 (18)	6.39 (3.71–11.02)	< 0.001
**Urine culture positive**	30 (7)	19 (27)	49 (10)	4.55 (2.39–8.66)	< 0.001
**Urine dipstix positive**	55 (14)	33 (46)	88 (18)	5.48 (3.18–9.43)	< 0.001
**Severe sepsis**	2 (0.5)	5 (7)	7 (1)	15.26 (2.90–80.27)	0.001
**Septic shock**	1 (0.2)	4 (6)	5 (1)	24.1 (2.7–219.0)	0.002

In a subgroup of SIRS and non-SIRS patients we compared patients with urine dipstix positive and negative for infection (Tables 3 and 4). Urine dipstix data was available in 72/99 SIRS patients (39 had negative and 33 had positive dipstix for infection) and 411/550 non-SIRS patients (356 had negative and 55 had positive dipstix for infection). Notably, 72% of SIRS patients with negative urine dipstix were discharged home compared to 21% of positive urine dipstix patients (*p* < 0.001). In comparison 88% of non-SIRS patients with negative urine dipstix was discharged home compared to 47% with positive urine dipstix. We evaluated urine dipstix, as this is a readily available test for decision-making in the ED about patient disposition, as urine culture typically is available 48–72 h later. In the non-SIRS patients with positive urine dipstix 52% underwent ureteral stenting compared to 10% with negative urine dipsitx. In our study more number of SIRS patients with dipstix negative for infection had ureteral stenting compared to non-SIRS patients (*p* < 0.05) also suggesting SIRS was an independent predictor of patients going to the OR with or without UTI. This was also confirmed by multivariate analysis. Table 5 represents patients who revisited ER and those who did not. It shows presence of SIRS was an independent risk factor (odds ratio 2.9) for revisit to the ED.

**Table 3:** SIRS patients with and without urinary infection

	SIRS (dipstix negative)	SIRS (dipstix positive)	***P***-value
No. patients	39	33	
Age (years)	42 (22–73)	46 (23–94)	0.545
Male (%)	28 (72)	9 (27)	< 0.001
Temp. (Celsius)	37 (36–38)	37 (36–39)	0.070
Heart Rate (beats/min)	100 (59–136)	109 (59–153)	0.016
Resp. Rate (breaths/min)	21 (16–32)	20 (15–36)	0.398
WBC # (cells/mm^3^)	14 (7–32)	15 (6–41)	0.810
Stone Size (mm)	4 (2–7)	7 (2–30)	0.136
D/C’d Home	28 (72)	7 (21)	< 0.001
To OR on 1st ED visit	9 (23)	24 (73)	< 0.001
Readmitted	3 (8)	2 (6)	1.000

**Table 4:** Non-SIRS patients with and without urinary infection

	Non-SIRS (dipstix negative)	Non-SIRS (dipstix positive)	***P***-value
No. patients	356	55	
Age (years)	41 (16–86)	44 (19–91)	0.195
Male (%)	229 (64)	18 (33)	< 0.001
Temp. (Celsius)	37 (36–37)	37 (36–38)	0.135
Heart Rate (beats/min)	77 (23–120)	84 (52–142)	0.025
Resp. Rate (breaths/min)	18 (10–28)	19 (16–24)	0.364
WBC # (cells/mm^3^)	10 (2.5–159)	10 (2–27.5)	0.738
Stone Size (mm)	4.5 (1–17)	6 (2–26)	< 0.001
D/C’d Home	315 (88.5)	26 (47)	< 0.001
To OR on 1st ED visit	35 (10)	29 (53)	< 0.001
Readmitted	9 (2.5)	1 (2)	1.000

**Table 5:** Univariate and multivariate analysis of factors for revisit to the ED

	Revisit to ED = N	Revisit to ED = Y		Multivariable Model			
	Univariate Model						
Variable	Mean (SD) or N (%)	Mean (SD) or N(%)	***p***-value	Odd’s Ratio	95 Lower	95 Higher	***p***-value
N	468	15					
Age	42 (16–94)	46 (18–70)	0.187	1.03	1.00	1.04	0.021
Male	277 (59)	7 (46)	0.426	0.50	0.26	0.54	< 0.001
SIRS	67 (14)	5 (33)	0.057	2.90	2.65	8.09	< 0.001
Sepsis	1 (0.1)	7 (1.5)	1.000	2.00	2.20	121.08	0.002
Stone Size	5 (1–30)	5 (3–13)	0.470	1.07	0.98	1.16	0.122
Temp.	37 (36–40)	37 (36–40)	0.830	1.10	1.31	3.11	0.142
Heart Rate	82 (23–153)	90 (58–116)	0.084	1.03	1.01	1.04	< 0.001
Respiratory Rate	19 (10–36)	18 (16–24)	0.370	1.07	0.98	1.16	0.122
WBC	11 (2–159)	11 (7–17)	0.849	1.06	1.08	1.23	0.121

## Discussion

In our tertiary hospital ED there was a 15% incidence of SIRS in the above population. Obstructing stones commonly result in ED visits but the majority of these patients are discharged home. Of the patients that require urgent renal drainage, many are for UTI or uncontrolled pain. There is no debate that patients with sepsis from an obstructing stone require emergent renal drainage. However, in a patient with an obstructing stone in the presence of SIRS, it is unclear what would be the patient outcome or disposition. Reports that biomarkers such as presepsin [7–9] and urine NGAL [10] improve the prognostic sensitivity of SIRS. However, these tests are experimental, costly and not commonly available in the ED in a timely manner.

Nadler et al. highlighted the stone burden on hospital admission rates and cost analysis of patients presenting with obstructing stones to the EDs across the United States [1]. Using a nationwide emergency department data sample they estimated an average of 1.2 million patients per year visiting ED yearly from 2006 to 2009 due to stones with a 19% hospital admissions. They identified infection and diabetes to be highly predictive of the need for admission on multivariate analysis. Theakston and co-authors identified stone size ≥5 mm, nitrites on urine dipstix, age > 50 years, and proximal ureteric stone as risk factors for urologic intervention within 3 months of visit to the ED. [2] Similarly, Papa et al. in addition to stone location and size, pain score upon discharge from ED was associated with urologic intervention within 1 month after initial presentation [11]. In our study apart from SIRS as a risk factor for urgent intervention and ED revisit, we found UTI on dipstix, age, presence of fever, elevated white cell count as other risk factors with OR > 1 (Table 2).

More recently the third international consensus for sepsis and septic shock updated the definitions of sepsis and septic shock [12]. The task force uses the qSOFA (quick Sequential Organ Failure Assessment) criteria RR > 22, altered mentation, systolic ≤ BP100. In the above study, qSOFA was better than SIRS criteria in the intensive care settings but similar to SIRS in an outside the ICU setting in predicting hospital mortality. The task force stresses that SIRS criteria still remain useful for the identification of sepsis. A more recent study, where the authors did a meta-analysis comparing the association of qSOFA and SIRS in predicting patient mortality in an ED setting [5]. Their results showed that both qSOFA score of ≥2 and a SIRS score of ≥2 were strongly associated with mortality in ER patients with infections. They showed qSOFA had higher specificity (88%) but lower sensitivity (42%), compared to SIRS, which had higher sensitivity (81%) and lower specificity (41%). The authors concluded that both are important at this time and that qSOFA cannot replace the use of SIRS in the ED setting until the sensitivity of qSOFA is improved.

Yamamoto et al. revealed that patient age (median 74 years) was an independent risk factor for the development of septic shock from an obstructing stone [13]. This corroborates our findings that advanced age increased the risk for requiring surgery (*p* = 0.026) (Table 2). Friedlander and colleagues reported that for every point increase in WBC count and pulse resulted in a 7 and 3% increase in sepsis, respectively [14]. The development of SIRS after ureteroscopy for stone disease has been reported but the role of SIRS in patients with obstructive urolithiasis presenting to the ED has not been studied [15].

To our knowledge there is no previously reported study evaluating the role of SIRS in obstructing stone patients presenting to the ED. A vast majority (77%) of our patients presenting to ED with an obstructing stone were discharged. However, SIRS patients were less likely (45%) to be discharged home compared to non-SIRS (83%) (*p* < 0.01). This may be because more number of SIRS patients had associated UTI and also other factors such as inadequate pain control might have contributed to this. Also, patients without infection, the presence of SIRS can be a reaction to inflammation, pain or other factors. These non-infective factors may have contributed to SIRS and the need for urgent renal drainage. Patient with SIRS who were discharged from the ED were more likely to revisit the ED (7%) compared to patients with no SIRS (2%, *p* = 0.05). In our cohort, patients with SIRS had higher incidence of UTI (49%)(on urine dipstix and culture) compared to those who did not have SIRS (13%), *p* < 0.01). However, patients with SIRS who were discharged from the ED with negative urine dipstix for infection had higher incidence of ED revisits compared to patients with no SIRS and had negative urine dipstix (Tables 3 and 4). Also, patients with SIRS had a higher likelihood of going into severe sepsis, 7% compared to 0.5% with no SIRS; albeit patients’ numbers were small. It is important to note that the judgment whether to drain the kidney or not should not be based solely on positive or negative urine dipstix but to be combined with other clinical factors including the presence of fever or chills, high white cell count etc.

Our hypothesis that SIRS impacts the disposition of patients including surgical intervention with obstructive urolithiasis was confirmed by this observational study. In the multivariate analysis SIRS was an independent factor associated with increased urgent surgical intervention and ED revisit. As our study was retrospective in nature, we could only conclude from our findings that SIRS is an independent risk factor for urgent drainage and revisits to the ED. The number of patients with critical illness (sepsis or septicemia) in our study group was very small and as many of our patients with SIRS had intervention, thereby preventing progression to severe sepsis or septic shock, it is difficult to conclude from the above study that SIRS is a predictor of critical illness in stone patients. Nevertheless, presence of SIRS should be an important factor in decision-making regarding patient disposition.

Limitations of our study include retrospective study design with lack of documentation of pain through a validated pain scale; hence, it is difficult to estimate the impact of pain on the patient disposition. However, all our patients had pain who had intervention and who revisited the ED with or without SIRS. We did not have urine cultures on all patients as many were discharged if the urine dipstix showed no infection. Also, some patients may have followed up at a different hospital following discharge from our ED, nevertheless as our data compared SIRS and non-SIRS patients, we presume the readmission percentage rates at other hospitals may be comparable to our center. Also, we did not have information on the stone type in the majority (> 95%) of our study population, we are not sure whether the stone type can influence which patients had SIRS with or without infection.

## Conclusions

SIRS was present in only 15% patients seen in our emergency department with obstructive urolithiasis. Presence of SIRS was indicative of increased need for urgent renal drainage with ureteral stenting and ED revisits. Although patients with SIRS had increased rate of associated UTI, SIRS was an independent risk factor for urgent urologic intervention with or without the presence of urinary infection. Presence of SIRS is one other risk factor to be considered during the early evaluation of patients presenting to the ED with obstructive stone colic while making decisions about treatment. Also, patients discharged who had SIRS but no urologic intervention had increased ED revisits. Revisits to ED may be minimized if a timely urology consultation as quickly as possible is made possible for patients with SIRS who were discharged from ED.

## Data Availability

The datasets generated and/or analyzed during the current study are not publicly available as it is in our database computer in our research office but are available from the corresponding author on reasonable request.

## Abbreviations

SIRS: Systemic Inflammatory Response Syndrome
ED: Emergency Department
qSOFA: quick Sequential Organ Failure Assessment)
STROBE: The Strengthening the Reporting of Observational Studies in Epidemiology

## References

[1] Eaton S, Cashy J, Pearl J, Stein D, Perry K, Nadler R (2013). Admission rates and costs associated with emergency presentation of urolithiasis: analysis of the Nationwide emergency department sample 2006-2009. J Endourol.

[2] Yan JW, McLeod SL, Edmonds ML, Sedran RJ, Theakston KD (2015). Risk factors associated with urologic intervention in emergency department patients with suspected renal colic. J Emerg Med.

[3] Bone RC, Balk RA, Cerra FB (2009). Definitions for sepsis and organ failure and guidelines for the use of innovative therapies in sepsis. The ACCP/SCCM Consensus Conference Committee. American College of Chest Physicians/Society of Critical Care Medicine. Chest.

[4] Kaukonen KM, Bailey M, Pilcher D, Cooper DJ, Bellomo R (2015). Systemic inflammatory response syndrome criteria in defining severe sepsis. N Engl J Med.

[5] Jiang J, Yang J, Mei J, Jin Y, Youjin L (2018). Head-to-head comparison of qSOFA and SIRS criteria in predicting the mortality of infected patients in the emergency department: a meta-analysis. Scand J Trauma Resusc Emerg Med.

[6] von Elm E, Altman DG, Egger M, Pocock SJ, Gøtzsche PC, Vandenbroucke JP (2007). The Strengthening the Reporting of Observational Studies in Epidemiology (STROBE) statement: guidelines for reporting observational studies. Lancet.

[7] Hou YS, Wang H, Chen H, Wu LF, Lu LF, He Y (2015). Pathfast presepsin assay for early diagnosis of systemic inflammatory response syndrome in patients with nephrolithiasis. Biomed Res Int.

[8] Ulla M, Pizzolato E, Lucchiari M (2013). Diagnostic and prognostic value of presepsin in the management of sepsis in the emergency department: a multicenter prospective study. Critical Care.

[9] Shozushima T, Takahashi G, Matsumoto N, Kojika M, Okamura Y, Endo S (2011). Usefulness of presepsin (sCD14-ST) measurements as a marker for the diagnosis and severity of sepsis that satisfied diagnostic criteria of systemic inflammatory response syndrome. J Infect Chemother.

[10] Zhu W, Liu M, Wang GC, Che JP, Xu YF, Peng B, Zheng JH (2014). Urinary neutrophil gelatinase-associated lipocalin, a biomarker for systemic inflammatory response syndrome in patients with nephrolithiasis. J Surg Res.

[11] Papa L, Stiell IG, Wells GA (2005). Predicting intervention in renal colic patients after emergency department evaluation. CJEM..

[12] Singer M, Deutschman CS, Angus D (2016). The third international consensus definitions for Sepsis and septic shock. JAMA..

[13] Yamamoto Y, Fujita K, Nakazawa S (2014). Clinical characteristics and risk factors for septic shock in patients receiving emergency drainage for acute pyelonephritis with upper urinary tract calculi. BMC Urol.

[14] Freidlander J, Jaimes, Garces J, Cuervo J, Ramirez F, Ramirez J, Vargas A (2003). The systemic inflammatory response syndrome (SIRS) to identify infected patients in the emergency room. Intensive Care Med.

[15] Southern JB, Higgins AM, Young AJ, Garg T (2019). Risk factors for post-operative fever and SIRS for stone disease. J Endourol.

